# Predictors of Difficult Ultrasound-Guided Transversus Abdominis Plane Blocks

**DOI:** 10.7759/cureus.18445

**Published:** 2021-10-02

**Authors:** Kyle Bellamy, Bryan J Hierlmeier, Oscar A Alam Mendez, Kenneth Oswalt, Tom Stockman

**Affiliations:** 1 Anesthesiology, University of Mississippi Medical Center, Jackson, USA; 2 Engineering, Los Alamos National Laboratory, Los Alamos, USA

**Keywords:** regional anesthesia, ultrasound guidance, ultrasound guided blocks and vascular access, ultrasound, anesthesia, muskuloskeletal ultrasound

## Abstract

Background

Fascial plane blocks are a valuable and important aspect of patient care. However, nerve blocks sometimes present with a technical difficulty that can lead to upsetting the operating room schedule, cause discomfort to the patient, or lead to inadequate block. Potential predictors of this difficulty were evaluated.

Methods

In a single-blind study, ultrasound image quality was evaluated on a grading metric, and its correlation with several factors that could potentially impact the difficulty of a procedure, including age, BMI, weight, length of surgery, IV fluids, and pre- vs postoperative block, was assessed.

Results

No correlation was found between any of our anesthetic, patient, or surgical factors, and the resulting image quality.

Conclusion

The study population was limited compared to our initial goals. We found no correlation between studied variables and image quality, but confounding factors that may affect image quality have not been ruled out.

## Introduction

Regional anesthesia is a key tool in patient care in both the operative and nonoperative settings. It plays a role in pain management and as a primary anesthetic. It also has the potential to play a role in short- and long-term outcomes, such as the incidence of phantom limb pain or length of hospital stay [1]. Ultrasound-guided regional anesthesia has become the preferred standard over landmark-guided or nerve stimulator-guided approaches alone [2]. However, the provision of care to patients via regional anesthesia requires the coordination of several factors.

The purpose of the block, the patient's need for pain control, and the mode of anesthetic administration are all examples of factors that play a role in optimal patient care. We should also consider case delays, staffing, and patient satisfaction.

Temporary inability to move an arm due to a nerve block may be more distressing than pain from the procedure. Communications with surgeons and operating room (OR) staff, as well as a supplied and dedicated block area are vital to adequate timing for the block to avoid case delays or prolonged turnovers [3]. However, any given block may present with a technical difficulty that can slow down the OR schedule. This may cause undue distress to the patient if being performed pre-induction or post-emergence. We hypothesize that various surgical, anesthetic, and patient factors may impact the technical difficulty of ultrasound-guided regional techniques.

## Materials and methods

**Figure 1:**
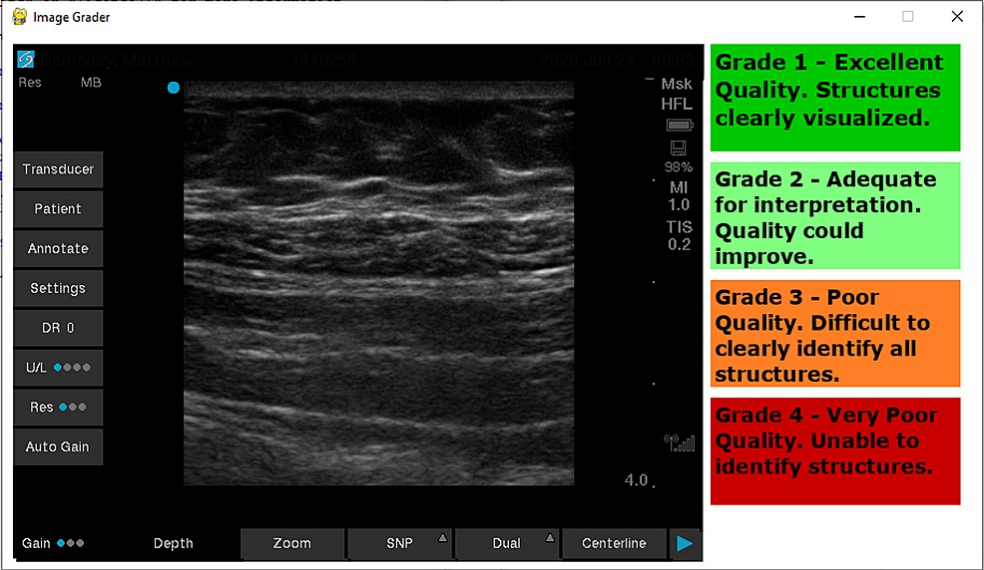
Python executable

We conducted a study to review factors that may be predictive of difficult regional anesthesia. Our dependent variable for difficulty was image quality. Our independent variables were operative time (pre vs post), operative length (minutes), weight (kg), BMI, age (years), and IV crystalloid administered (liters). A grade scale for image quality was created, as shown in Figure 1. IRB approval was obtained, and patients provided consent for the study. A total of 28 patients were enrolled. The exclusion criteria are illustrated in Table 1.

**Table 1:** Study criteria TAP, transversus abdominis plane

Inclusion Criteria	Exclusion Criteria
Adult patients greater than 18 years of age	Emergency surgery
Scheduled for open abdominal surgery	Surgical patients who do not receive abdominal wall closure
	Patients getting TAP block prior to surgery
	Patients with abdominal surgery six weeks prior
	Patients without pre- and postoperative images

Due to exclusion criteria, 12 patients were excluded leaving 16 patients whose ultrasound images and relevant data we collected. Images were gathered and designated as preoperative and postoperative based on the time signature saved to the image. A script was then written to display images at random, as shown in Figure 1.

Ultrasound images were obtained by two anesthesiologists trained in ultrasound and regional anesthesia. Protected health information was removed following image collection. The image reviewer selected was an expert in pain management and regional anesthesia and was not involved in obtaining images. The reviewer viewed each image and assigned it a grade. Once images were graded, the data were retrieved and decrypted by investigators.

## Results

**Figure 2:**
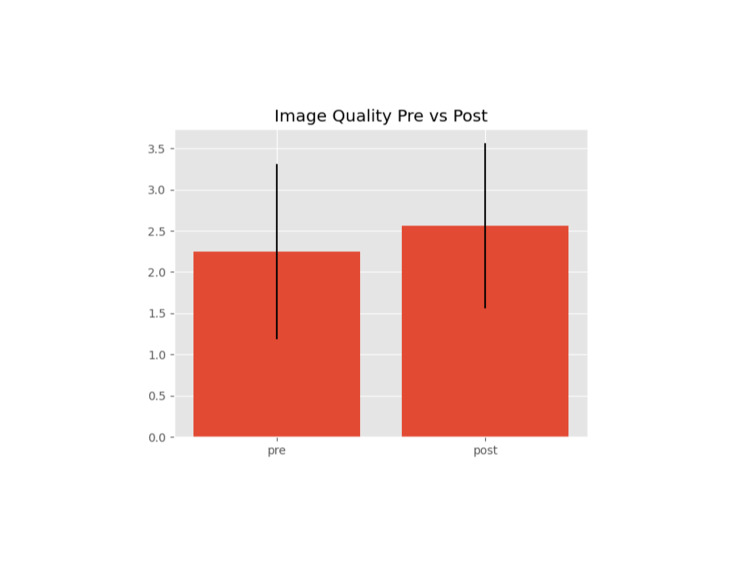
Average image quality and standard deviation for preoperative images

**Figure 3:**
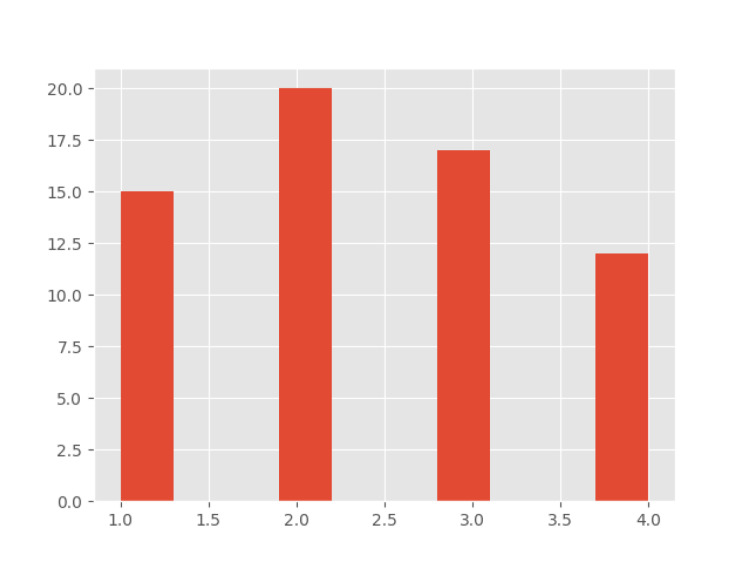
Histogram for the distribution of image quality

A total of 64 images were obtained. Image quality data were deciphered using the computer program Python, which assigned the graded image to the appropriate patient. Potentially relevant case data were also obtained from the electronic medical records to review other potential factors that may influence outcomes. Data reviewed included patient age, BMI, and weight, IV crystalloid administered, length of surgery. and image quality. First image quality was evaluated, as shown in Figure 2, and image quality was compared between pre- and postsurgery, as shown in Figure 3.

**Figure 4:**
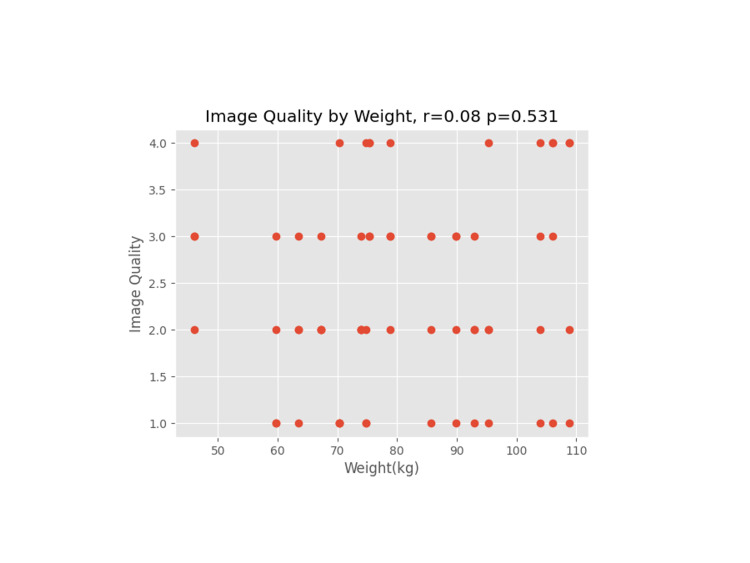
Image quality versus patient weight (in kg)

**Figure 5:**
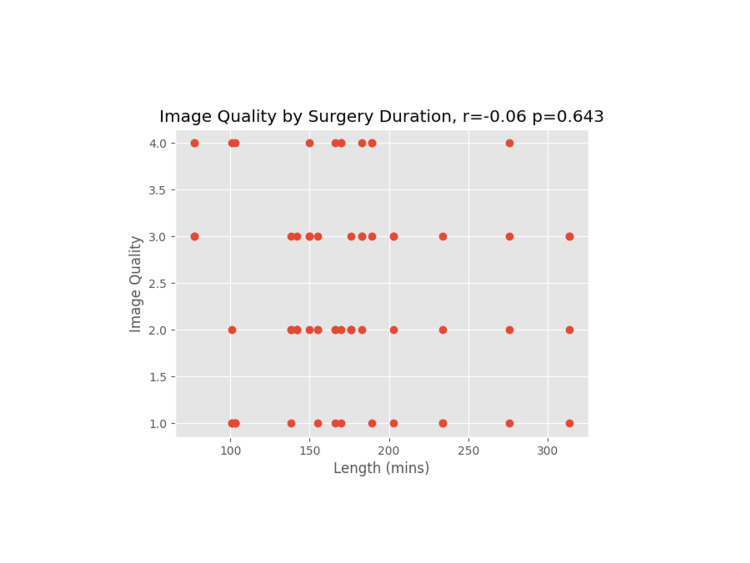
Image quality versus surgery duration (in minutes)

**Figure 6:**
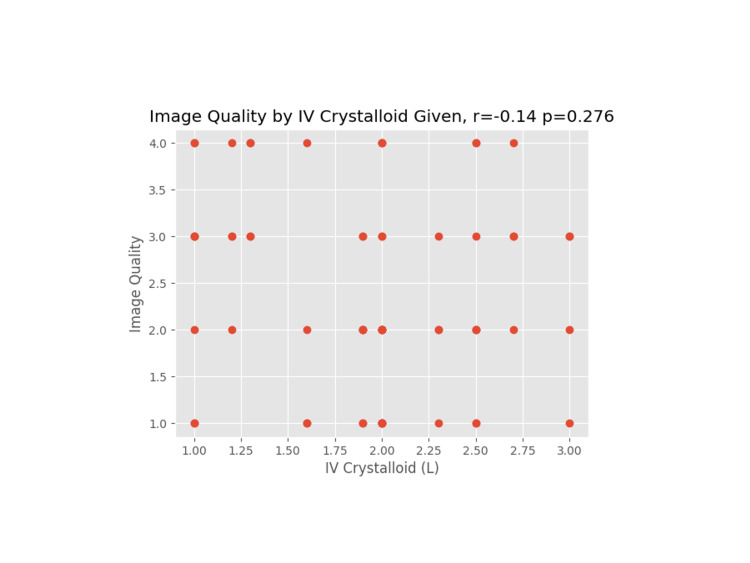
Image quality versus IV crystalloid administration

**Figure 7:**
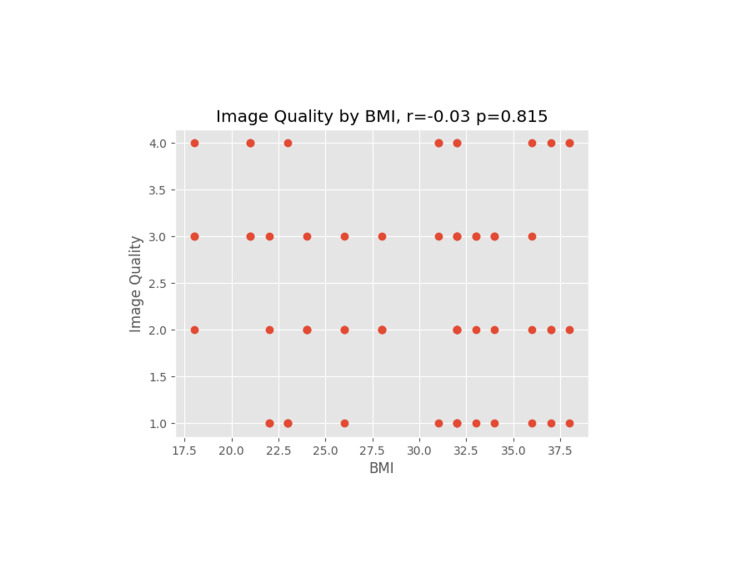
Image quality versus BMI

**Figure 8:**
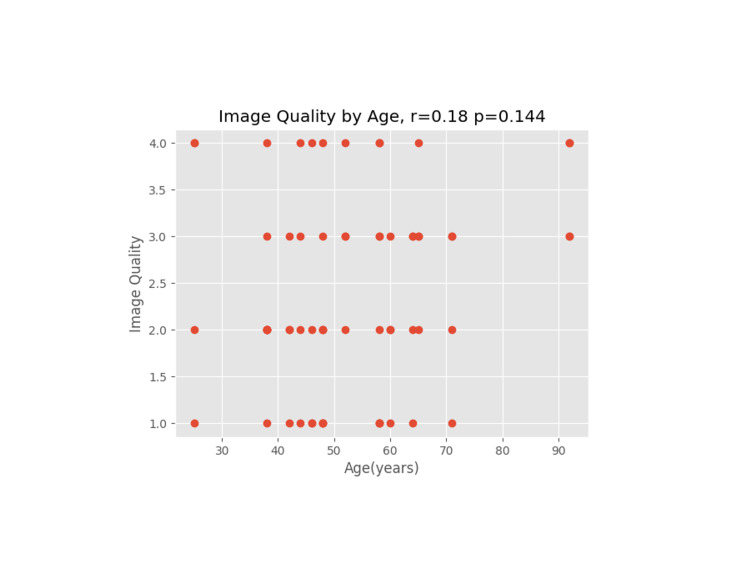
Image quality versus age (in years)

Grade 2 was the most common grade assigned to an image. Evaluating image grades for all study patients preoperatively showed an average image score of 2.25 with a standard deviation of 1.060. Postoperatively, the average score was 2.56 with a standard deviation of 0.998. Scatter plots of image quality versus other potential factors such as patient weight, surgery duration, IV fluid administration, BMI, and age are illustrated in Figures 4-8.

A Pearson coefficient was used on each plot to determine the correlation coefficient and p-value for the comparative data sets.

## Discussion

Regional anesthesia provides many potential benefits. Depending on patient status, surgical intervention, and the block used, studies have shown improvements in respiratory complications, acute or chronic pain, opioid use, and postoperative nausea and vomiting [4,5]. However, accurately communicating with all teams involved is essential for timely intervention to provide patients with the best relief. Some studies have indicated that a preoperative block for an extremity amputation may reduce the incidence of phantom limb pain [6]. For transversus abdominis plane (TAP) blocks specifically, there is no clear evidence of increased effectiveness if done preoperatively versus postoperatively [7,8]. Difficulty in performing the procedure may impact overall procedure time, leading to potential OR delays, prolonged OR time, or prolonged patient discomfort if performed post-emergence. OR delays are a significant source of concern not only for emergency availability but also for cost as one minute in the OR can cost up to $36 [9]. We reviewed several potential factors that could impact the difficulty of a TAP block using Image quality as a standard scale. Initially, we reviewed image quality in patients with images taken prior to surgery and after surgery, with one being a textbook image of muscular planes on ultrasound and four being very difficult to identify. On average, preoperative images scored 2.25 and postoperative images scored 2.56. Standard deviation for both values was also high. Initial data appear to show very little indication that timing of TAP blocks plays a role in the difficulty of the procedure as measured by image quality (other potential sources for difficulty).

We proceeded to evaluate other patient and operative factors that might affect image quality. Weight/BMI is often a concern for block difficulty due to excess tissue that may affect the image or needle manipulation, especially as increased depth on an ultrasound necessitates a less clear image [10]. For the rest of the data reviewed, a correlation coefficient (r) and p-value (p) were calculated. An absolute value of r close to 1 indicates data that closely correlate, whereas the p-value indicates the probability of a correlation showing in an uncorrelated data set. None of the hypothesized data points (weight, BMI, IV crystalloids, age, and length of surgery) had a significant correlation to image quality.

Of note, the overall size of this study was limited due to a larger quantity of laparoscopic and robotic procedures versus open procedures than predicted. Inadequate ultrasound availability also inhibited patient enrollment. In addition to increasing size, there are still several other factors that could be worth studying. There are several approaches to a TAP block, and the best TAP block for a patient is typically determined by the location of surgery. All of our images were obtained as a lateral approach regardless of upper versus lower versus unilateral procedure [11]. Lastly, getting input from more specialists might help determine the validity of the grading metric used.

## Conclusions

Based on the data collected, there is no clear predictor for when ultrasound imaging will be more or less challenging. Age, weight, BMI, length of surgery, IV fluids administered, and operative changes do not seem to impact the image obtained of relevant planes. While this does not necessarily help guide timing of regional anesthesia for patient care, considerations to provide timely and effective care to the patient should still be reviewed and optimized through institutional guidelines.
